# Exercise-induced Brugada pattern and ventricular tachycardia during Capecitabine treatment

**DOI:** 10.1186/s40959-022-00146-x

**Published:** 2022-11-19

**Authors:** George B. Strobel, Shiva P. Ponamgi, Attila Roka, Ahmed S. A. Aboeata

**Affiliations:** 1 Creighton University School of Medicine, Omaha, NE USA; 2 Department of Cardiology, Creighton University School of Medicine, Omaha, NE USA

## Abstract

We report the case of a 59-year-old female patient with no previous cardiovascular disease treated for Breast cancer with Capecitabine. Shortly after starting treatment, she developed recurrent angina. An exercise stress echocardiogram was performed, which induced a type 1 Brugada pattern 12 s of a non-sustained pleomorphic ventricular tachycardia ensued.

## Capecitabine mechanism

5-Fluorouracil (5-FU) was discovered in 1957 and used as a chemotherapeutic drug to treat a variety of cancers. It is a commonly used treatment for advanced or metastatic gastrointestinal and breast cancers. The molecule inhibits Thymidylate synthase, thus inhibiting DNA synthesis due to a lack of the crucial substrate Thymine. Capecitabine (brand-name Xeloda) is an oral prodrug form of 5-FU with enhanced tumor selectivity and less cardiotoxicity. The accepted mechanism of action is that the intracellular Thymine phosphorylase cleaves Capecitabine to form 5-FU. It can be further cleaved into a cardiotoxic metabolite called Fluoroacetate, which can induce myocardial infarction, heart failure, or arrhythmias. One study found capecitabine-related cardiotoxicity in 5.9% of patients, and severe cardiotoxicity in 2.3% of patients. Combination treatment with capecitabine, oxaliplatin, and bevacizumab was associated with the highest risk of cardiotoxicity [1] (Kwakman et al.).

## Case report

**Fig. 1:**
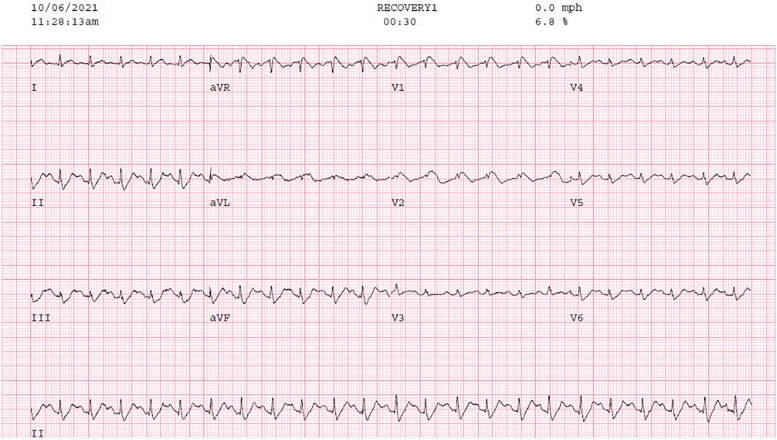
[EKG showing suspected type 1 Brugada pattern during Exercise stress test] The inferoapical STD however is not typical for Brugada and may be a hint ischemia – mirror changes for the septal ST elevation

**Fig. 2:**
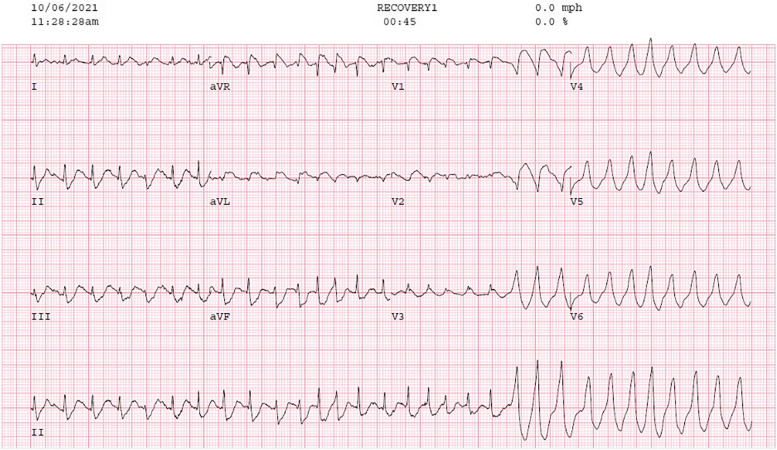
[EKG showing Initiation of NSVT] Irregular wide QRS tachycardia with fusion beats. Differential diagnosis would be pre-excited atrial fibrillation; however, no delta wave is seen at baseline or with PACs

A 59-year-old Caucasian female, with no significant prior cardiac history and who never smoked, was diagnosed with, ER negative, PR negative, HER2/neu 2+ equivocal, grade 3 right breast invasive ductal carcinoma. The patient received 4 cycles of anthracycline chemotherapy, a two-month course of Docetaxel, Trastuzumab, and Pertuzumab, right lumpectomy, and right breast radiation (5256 cGy over 20 fraction). Due to continuing neoplastic proliferation, Capecitabine treatment was added, planned for 2500 mg PO twice daily for 1 month. One day after starting the medication, the patient developed exertional angina-like chest pain and intermittent shortness of breath. An exercise stress echocardiogram was ordered to further evaluate her symptoms during which she developed a type one Brugada pattern (Fig. 1) at a heart rate of 161 bpm after achieving 7.7 METS, which deteriorated to a recurrent and non-sustained pleomorphic ventricular tachycardia (VT) (Fig. 2) that spontaneously resolved with discontinuation of the stress test. Prior to the stress test, she had a normal EKG. The patient underwent a coronary angiogram which showed normal coronary arteries. Genetic testing was negative for any inherited arrhythmia syndromes. Subsequently, a cardiac MRI with late gadolinium enhancement was done, which showed no evidence of myocardial scarring (late gadolinium enhancement) or any structural abnormality. A procainamide challenge test was done, and we were unable to induce the Brugada pattern or any VT. The picture was strongly suggestive of coronary vasospasm being the etiology of her presentation, so she was started on diltiazem and isosorbide mononitrate. After a discussion with the oncologist, capecitabine was deemed to be a necessary drug for her breast cancer treatment with no safer alternatives, so it was resumed with close monitoring in the Cardio-Oncology clinic. The patient had continuous ambulatory cardiac monitoring for 1 month with no significant sustained arrhythmias while receiving capecitabine. Due to progressive disease, Capecitabine treatment with the concomitant use of the calcium channel blocker and the nitrate was successfully finished with no recurrent angina or arrhythmias.

## Discussion

This case report documents the unmasking of a Brugada type I EKG pattern and induction of pleomorphic VT during exercise stress testing probably due to Capecitabine-induced coronary vasospasm and myocardial ischemia in a patient with no previous cardiac history while on Capecitabine therapy. Capecitabine cardiotoxicity has been reported ever since its FDA approval in the 1990s. The active ingredient of the pro-drug Capecitabine is 5-FU. The proposed mechanism of 5-FU cardiotoxicity is direct injury to the myocardium or coronary arteries which induces vasospasm. This could then lead to altered flow and possible thrombogenesis [1]. Our patient started having angina the day after capecitabine was started and had no evidence of obstructive coronary artery disease on angiography and no structural cardiac abnormalities on cardiac MRI.

The Brugada brothers introduced Brugada syndrome in 1992, as a “Right precordial ST-elevation followed by a negative T-wave and a high incidence of ventricular fibrillation (VF) in the absence of structural heart disease [2].” Brugada syndrome is normally an inherited syndrome. To our knowledge, the patient has no family history of Brugada syndrome, and the pattern has not returned after starting Diltiazem and Isosorbide mononitrate. The patient underwent procainamide challenge and genetic testing, both of which were negative for Brugada syndrome, suggestive of a Brugada pattern being induced by myocardial ischemia due to coronary vasospasm caused by Capecitabine.

Vasospastic angina (VSA) is a less common form of angina compared to the predominant angina caused by obstructive coronary artery disease. VSA is most likely caused by hypercontractility of coronary smooth muscle, endothelial dysfunction, magnesium deficiency, low-grade inflammation autonomic dysfunction, and oxidative stress [3], all factors of vascular regulation. Picard et al. suggests that VSA, “is underdiagnosed and provocative tests are rarely performed [3].” This is the case because VSA can cause angina in patients with or without previous cardiac history, making it especially difficult to distinguish in the case with a previous cardiac history. The diagnosis of VSA includes coronary angiography with provocative stimuli such as acetylcholine or ergonovine, response to nitrates, and transient ischemic patterns on EKG [3]. Unfortunately, we were unable to perform a coronary provocative test on our patient. While transient ischemic changes like ST segment elevation ≥0.1 mV, ST-segment depression ≥0.1 mV or inverted U waves seen during anginal episodes are suggestive of VSA QRS changes and atypical changes may be noted if a large amount of the myocardium or the conduction system is involved [4]. VSA is commonly treated with calcium channel blockers and Nitrates, although calcium channel blockers are also implicated in the provocation of a Brugada EKG pattern [5, 6], we have not noticed any recurrence of the pattern in our patient after initiation of treatment with Diltiazem and Isosorbide mononitrate, The patient has continued Diltiazem and Isosorbide mononitrate to date, after finishing Capecitabine treatment.

Overall, a Brugada ECG pattern has been suggested to be a repolarization disorder [7], associated with increased vagal activity and withdrawal of sympathetic activity. One study was able to induce Brugada-like patterns in canines under the conditions of slow pacing, acetylcholine, and sodium channel blockade [8]. Although, there is no evidence that suggests that Capecitabine or other Fluorouracil drugs have sodium channel blocking effects. If a Brugada pattern is identified, it is likely an ischemic phenomenon. A few earlier case reports and case series reported exercise-induced syncope and ST elevations which were helpful in the unmasking of Brugada pattern in patients with genetically established Brugada syndrome [7]. They also concluded that exercise-induced ST elevations in patients with Brugada syndrome could be an independent predictor for future cardiac events portending a poorer prognosis and suggested that beta-blockers could be beneficial in this sub-group [9]. We find it interesting that the patient we report here with negative genetic testing for Brugada syndrome, had a pronounced Brugada pattern during exercise, the reverse of conventional knowledge on Brugada.

Capecitabine is an efficacious drug and brings great benefits to patients with its comparably favorable adverse effects [10]. Capecitabine Cardiotoxicity is often transient and reversible, although there are more severe cases reported [10]. In some patients despite the risk of cardiotoxicity, Capecitabine may remain the drug of choice. In these cases, re-challenging therapy may be tried with close monitoring. In the case of suspected Acute Capecitabine cardiotoxicity Kanduri et al. further suggested in cases of re-challenging therapy, pre-treating with Calcium channel blockers and Nitrates, giving Calcium channel blockers during therapy and continuing Calcium Channel blocker/Nitrate treatment after therapy [11].

## Conclusion

Capecitabine is an accepted treatment for breast and gastrointestinal cancers, two of the most common types of Cancer, therefore providers and patients will likely confront the cardiotoxic effects of treatment. Providers need to be aware of the Cardiotoxic risks of Capecitabine and how to evaluate and remediate them. We reinforce the need for patients undergoing treatment with Capecitabine to be followed for symptoms of angina, alongside careful attention to be given to its etiology.

## Data Availability

No data sets used, only figures taken from actual patient studies.

## References

[1] Kwakman JJ, Simkens LH, Mol L, Kok WE, Koopman M, Punt CJ (2017). Incidence of capecitabine-related cardiotoxicity in different treatment schedules of metastatic colorectal cancer: a retrospective analysis of the CAIRO studies of the Dutch colorectal Cancer group. Eur J Cancer.

[2] Hoogendijk MG, Opthof T, Postema PG, Wilde AA, de Bakker JM, Coronel R (2010). The Brugada ECG pattern: a marker of channelopathy, structural heart disease, or neither? Toward a unifying mechanism of the Brugada syndrome. Circ Arrhythm Electrophysiol.

[3] Picard F, Sayah N, Spagnoli V, Adjedj J, Varenne O (2019). Vasospastic angina: a literature review of current evidence. Arch Cardiovasc Dis.

[4] Beltrame JF, Crea F, Kaski JC (2017). International standardization of diagnostic criteria for vasospastic angina. Eur Heart J.

[5] Antzelevitch C, Pollevick GD, Cordeiro JM, Casis O, Sanguinetti MC, Aizawa Y, Guerchicoff A, Pfeiffer R, Oliva A, Wollnik B, Gelber P, 288 Circ Arrhythm Electrophysiol June 2010 Downloaded from http://ahajournals.org by on February 27, 2022.

[6] Bonaros EP, Burashnikov E, Wu Y, Sargent JD, Schickel S, Oberheiden R (2007). Loss-of-function mutations in the cardiac calcium channel underlie a new clinical entity characterized by ST-segment elevation, short QT intervals, and sudden cardiac death. Circulation..

[7] Yan GX, Antzelevitch C (1999). Cellular basis for the Brugada syndrome and other mechanisms of arrhythmogenesis associated with ST-segment elevation. Circulation..

[8] Nabauer M, Beuckelmann DJ, ÜBerfuhr P, Steinbeck G (1996). Regional differences in current density and rate-dependent properties of the transient outward current in subepicardial and subendocardial myocytes of human left ventricle. Circulation..

[9] Batra AS, Watson R, McCanta AC (2019). Exercise-induced syncope and Brugada syndrome. Ann Pediatr Cardiol.

[10] Wagstaff AJ, Ibbotson T, Goa KL (2003). Capecitabine: a review of its pharmacology and therapeutic efficacy in the management of advanced breast cancer. Drugs..

[11] Kanduri J, More LA, Godishala A, Asnani A (2019). Fluoropyrimidine-associated cardiotoxicity. Cardiol Clin.

